# Emerging Molecular Targets for the Treatment of Refractory Sarcoidosis

**DOI:** 10.3389/fmed.2020.594133

**Published:** 2020-11-24

**Authors:** Gonçalo Boleto, Matheus Vieira, Anne Claire Desbois, David Saadoun, Patrice Cacoub

**Affiliations:** ^1^AP-HP, Groupe Hospitalier Pitié-Salpêtrière, Department of Internal Medicine and Clinical Immunology, Paris, France; ^2^Centre de Référence des Maladies Auto-Immunes et Systémiques Rares, Centre de Référence des Maladies Auto-Inflammatoires et de l'Amylose, Bordeaux, France; ^3^Sorbonne Université, UPMC Univ Paris 06, UMR 7211, Inflammation-Immunopathology-Biotherapy Department (DHU i2B), Paris, France; ^4^INSERM, UMR_S 959, Paris, France; ^5^CNRS, FRE3632, Paris, France

**Keywords:** sarcoidosis, therapy, JAK inhibitors, interleukin-1, interleukin-6, granuloma

## Abstract

Sarcoidosis is a multisystem granulomatous disease of unknown origin that has variable clinical course and can affect nearly any organ. It has a chronic course in about 25% of patients. Corticosteroids (CS) are the cornerstone of therapy but their long-term use is associated with cumulative toxicity. Commonly used CS-sparing agents include methotrexate, cyclophosphamide, azathioprine, and mycophenolate mofetil. Twenty to forty percentage of sarcoidosis patients are refractory to these therapies or develop severe adverse events. Therefore, additional and targeted CS-sparing agents are needed for chronic sarcoidosis. Macrophage activation, interferon response, and formation of the granuloma are mainly mediated by T helper-1 responses. Different pro-inflammatory cytokines such as interleukin (IL)-8, IL-12, IL-6, and tumor necrosis factor-alpha (TNF-α) have been shown to be highly expressed in sarcoidosis-affected tissues. As a result of increased production of these cytokines, Janus kinase-signal transducer and activator of transcription (JAK-STAT) signaling is constitutively active in sarcoidosis. Several studies of biological agents that target TNF-α have reported their efficacy and appear today as a second line option in refractory sarcoidosis. Some case series report a positive effect of tocilizumab an anti-IL-6 monoclonal antibody in this setting. More recently, JAK inhibition appears as a new promising strategy. This review highlights key advances on the management of chronic refractory sarcoidosis. Novel therapeutic strategies and treatment agents to manage the disease are described.

## Introduction

Sarcoidosis is a multisystem granulomatous disease that can involve virtually any organ though the lungs and the lymphatics are the most commonly affected sites (1). The disease may remit spontaneously or upon treatment usually within the first 2–3 years after diagnosis but can have a chronic course in about 25% of cases (2). The exact cause of sarcoidosis is still not known. However, genetic susceptibility and environmental factors have been suggested as contributors of disease development (3, 4). Several studies strongly suggest that sarcoidosis might be the result of an exaggerated granulomatous reaction to a microbial-induced host response and persistent presence of antigens causing sarcoid lesions (5). Systematic treatment is often reserved for life-threatening organ involvement [i.e., severe interstitial lung disease, central nervous system (CNS), kidneys, liver, heart] or severe disabling functional symptoms such as skin disease, arthritis and bone disease or posterior uveitis (1). Corticosteroids (CS) remain the mainstay of treatment; however, their long-term use is associated with cumulative toxicity. Alternative therapies commonly used as CS-sparing agents include hydroxychloroquine, methotrexate, azathioprine and mycophenolate mofetil (6). About 10% of patients are refractory to these first and second-line therapies or develop adverse events necessitating additional CS-sparing targeted agents (7). Despite limited data on extra pulmonary manifestations, cyclophosphamide (CYC) has been successfully used for refractory CNS and cardiac disease (8–10). However, the known toxic and carcinogenic profile of CYC limits its use especially in young patients. In this setting, biological agents that target the tumor necrosis factor (TNF) have been introduced as a third-line therapy and have proved to be effective in a proportion of patients with severe/refractory sarcoidosis (11). Despite their excellent safety profile in other rheumatic conditions, TNF-α antagonists use showed to be less well tolerated with severe infections and malignancies being more frequent during sarcoidosis treatment (12). Hence, there is currently an unmet therapeutic need for a proportion of refractory or intolerant patients to TNF antagonists. Identifying new molecular targets for the treatment of refractory sarcoidosis should be a priority. Here, we review new therapeutic approaches for the treatment of refractory sarcoidosis with targeted biologic and synthetic agents.

**Figure 1:**
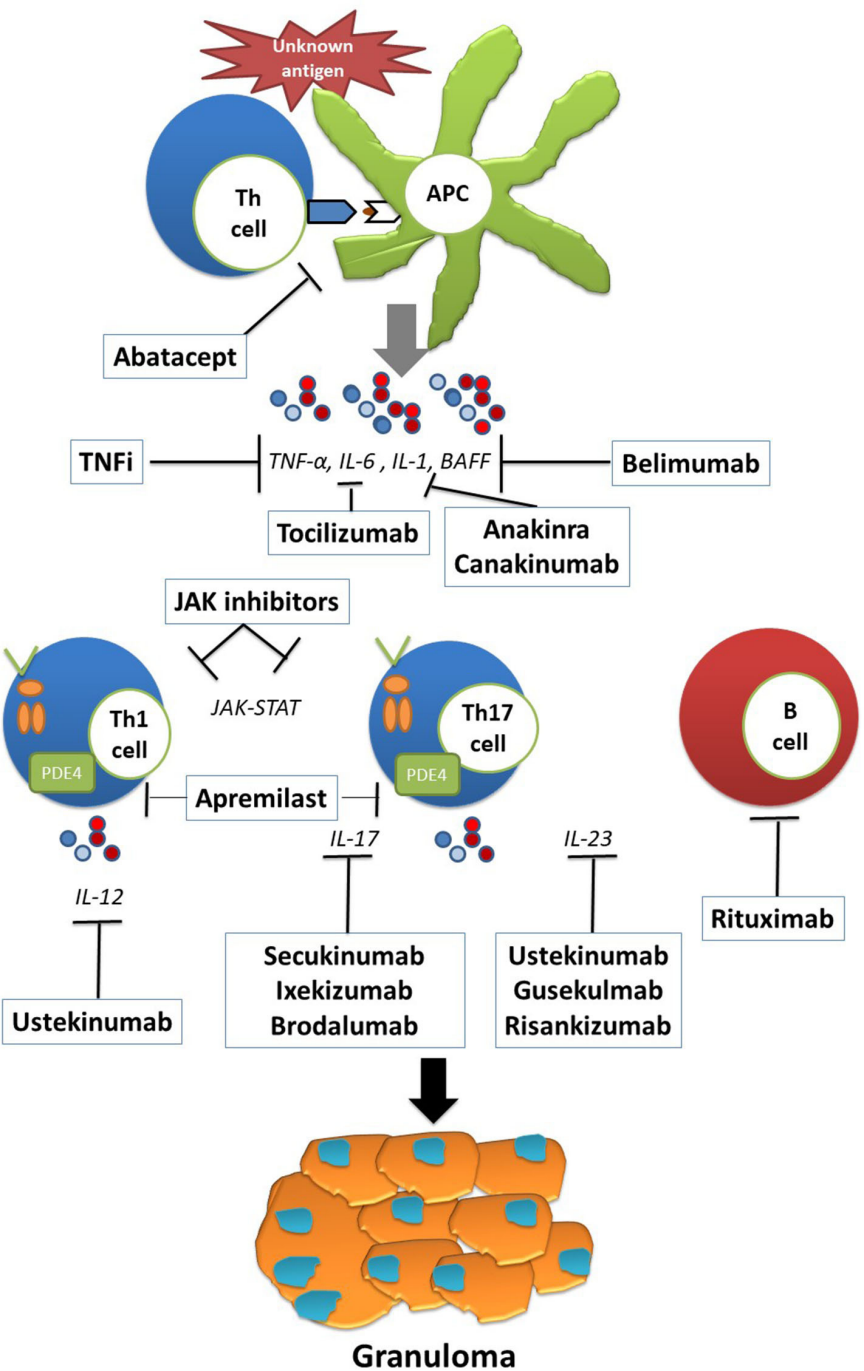
The pathophysiology and potential therapeutic targets of refractory sarcoidosis. Exposure to an unknown antigen leads to the activation and proliferation of T cells through antigen presenting cells (APCs). The release of proinflammatory cytokines such as IL-6, IL-1, and BAFF skews the immune response to Th1 and Th17 responses as well as B cell activation and proliferation. Persistent antigen presentation leads to granuloma formation and to the development of sarcoid lesions. Th, T-helper cell; APC, antigen presenting cell; IL, interleukin; BAFF, B lymphocyte stimulator; JAK, janus kinase.

A summary of the current data on the potential therapeutic targets in refractory sarcoidosis is available in Table 1. A focus on the reported cases of targeted biologic and synthetic agents (other than TNF inhibitors) use in sarcoidosis is available in Supplementary Table 1. Figure 1 illustrates the pathophysiology and potential therapeutic targets of refractory sarcoidosis.

**Table 1:** Current data on the potential therapeutic targets in refractory sarcoidosis.

**Target**	**Agents**	**Evidence**	**Primary endpoint**	**Ongoing clinical trials**
IL-6	Tocilizumab Sarilumab	Case-reports		Phase II (Sarilumab) (NCT04008069)
IL-1	Anakinra Canakinumab	Basic studies		Phase II (Canakinumab) for pulmonary sarcoidosis (NCT02888080) Phase II (Anakinra) for cardiac sarcoidosis (NCT04017936)
IL-17	Secukinumab Ixekizumab Brodalumab	Case-reports Phase II RCT (Secukinumab for non-infectious uveitis)	NA (only 4 patients)	None
IL-12/23	Ustekinumab (IL-12/23) Guselkumab (IL-23) Risankizumab (IL-23)	Phase II RCT (Ustekinumab for pulmonary and skin sarcoidosis)	Negative	None
CTLA4	Abatacept	Basic studies		STAR trial (NCT00739960) (the study has been terminated prematurely due to funding constraints)
B-cell	Rituximab Belimumab	Case-reports Open-label phase I/II trial (Rituximab for pulmonary sarcoidosis)	Negative	None
JAK	Ruxolitinib Tofacitinib Baracitinib Upadacitinib Filgotinib	Case-reports		Open-label trial (Tofacitinib) for pulmonary sarcoidosis (NCT03793439) Open-label trial (Tofacitinib) for cutaneous sarcoidosis (NCT03910543)
PDEA4	Apremilast	Open-label trial (cutaneous sarcoidosis)	Positive	Phase II/III open-label trial for cutaneous sarcoidosis (NCT00794274)

*IL, interleukin; RCT, randomized-clinical trial; NA, not evaluated; CTLA4, cytotoxic T-lymphocyte antigen 4; JAK, janus kinase; PDEA4, phosphodiesterase type 4*.

## Tumor Necrosis Factor-Alpha Inhibition

TNF-α plays a crucial role in the development of noncaseating granulomas in a variety of diseases. Previous studies showed that high levels of TNF-α from alveolar macrophages correlated with disease progression (13). Two randomized controlled trials have investigated infliximab, a chimeric monoclonal anti-TNF-α antibody, therapy in sarcoidosis and showed significant though modest improvement in lung function after 14 weeks of treatment (14, 15). Another randomized controlled trial showed significant improvement in extrapulmonary sarcoidosis as assessed by a the extrapulmonary physician organ severity tool (ePOST) in patients treated with infliximab (16). A large French nationwide multicentric retrospective study on 132 patients with refractory sarcoidosis showed TNF-antagonists to be efficient in about two-thirds of patients, despite higher rates of adverse events (11). Adalimumab, another monoclonal anti- TNF-α antibody, showed to be efficient in a randomized controlled clinical trial of 16 patients with skin sarcoidosis (17). These positive effects of TNF inhibition are supported by a high relapse rate after discontinuation of infliximab therapy in 47 patients with severe sarcoidosis (18). Despite the lack of high quality evidence, TNF inhibition can currently be considered as standard-of-care in severe cases of refractory sarcoidosis.

## Interleukin-6 Blocking

A subset of CD4^+^ effector T cell population called T helper 17 (Th17), which express a transcription factor known as retinoic acid-related orphan receptor (ROR)γι, has been described in sarcoid lesions (19). Active sarcoidosis patients have an increased Th17/T regulator (Treg) ratio in the peripheral blood and bronchoalveolar lavage fluid (BALF) that is reversed by immunosuppressive therapy (20). Interleukin-6 (IL-6) is a key pleiotropic cytokine. It induces the development of Th17 cells from naïve T cells together with transforming growth fractor β (TGF-β) and it inhibits anti-inflammatory Treg cells (21). Previous reports showed increased IL-6 levels in the BALF of sarcoidosis patients. Significant correlations were found between IL-6 levels and CD4^+^/CD8^+^ ratio, IL-8 and BALF neutrophil percentage (22–24). Another study reported increased IL-6 levels in the cerebrospinal fluid (CSF) of patients with neurosarcoidosis as compared to patients with multiple sclerosis or other inflammatory disorders. CSF concentration of IL-6 > 50 pg/mL was associated with a higher risk of relapse or progression of neurosarcoidosis (25). Genetic variations in the genes encoding IL-6 were preferentially upregulated in patients with severe and progressive sarcoidosis thus giving further evidence of the pathogenic role of this proinflammatory cytokine (26, 27). IL-6 is a potent up regulator of serum amyloid A protein (SAA), an acute phase reactant which has demonstrated a potential key role in the pathogenesis of sarcoidosis (28).

Currently, there are two humanized IL-6 receptor monoclonal antibodies [tocilizumab (TCZ) and sarilumab (SAR)] that have been approved for the treatment of different inflammatory rheumatic conditions. Despite robust evidence on the role of IL-6 in the pathogenesis of sarcoidosis, data on the use of anti-IL-6 agents in sarcoidosis are scarce. Previous case reports showed clinical improvement under TCZ in patients with chronic sarcoidosis associated with Castleman's disease (29) and Still's disease (30). In a case series of four patients with refractory chronic sarcoidosis the authors reported dramatic responses to TCZ with improved symptoms and organ function allowing steroid tapering (31). In a model of experimental uveitis, Yoshimura et al. investigated the role of IL-6 in the formation of refractory ocular inflammation (32). The authors showed that IL-6-deficient mice had reduced Th17 responses and ameliorated ocular inflammation. Using the same model of experimental uveitis, systemic administration of an anti-IL-6 receptor antibody also ameliorated ocular inflammation by suppressing Th17 responses. TCZ has shown a positive signal in the management of patients with ocular involvement (refractory uveitis, cystoid macular oedema) refractory to conventional immunosuppressive drugs (33, 34). Of note, despite these encouraging data, cases of paradoxal new-onset sarcoidosis during TCZ therapy must also be acknowledge. To our knowledge, four reports [three patients with rheumatoid arthritis (RA) (35–37) and one with giant cell arteritis (38)] have described cases of cutaneous and mediastinal sarcoidosis triggered by TCZ therapy. The exact mechanism for these paradoxical reactions is not known, but data on murine models of granulomatosis suggest that anti-IL-6 treatment might enhance TNF-α production contributing to granuloma formation (39, 40). Currently there is an ongoing clinical trial comparing the effectiveness and the safety of SAR in patients with glucocorticoid-dependent sarcoidosis (NCT04008069).

## Interleukin-1 Blocking

Interleukin-1 (IL-1) is a cytokine with potent pro-inflammatory properties that has been shown to be implicated in the pathogenesis of sarcoidosis (41). An imbalance between the levels of IL-1β and IL-1 receptor antagonist has been observed in the BALF of patients with pulmonary sarcoidosis (42, 43). Anakinra is a recombinant human IL-1 receptor antagonist that was firstly approved for the treatment of RA. It is currently used mostly for the management autoinflammatory conditions and difficult-to-treat gout (44, 45). We were unable to identify case reports or trials of anakinra use in the treatment of sarcoidosis. However, two cases of anakinra-induced sarcoidosis have been reported (46, 47). A phase 2 randomized controlled trial assessing the efficacy and safety of canakinumab, another IL-1 antagonist, in patients with pulmonary sarcoidosis (NCT02888080) has completed the recruitment process. The Interleukin-1 Blockade for Treatment of Cardiac Sarcoidosis (MAGiC-ART) trial (NCT04017936) is another ongoing phase 2 randomized-controlled trial evaluating anakinra for the treatment of cardiac sarcoidosis.

## Interleukin-17 Blocking

Recent investigations indicate that Th17 cells are key players in all stages of granuloma formation and are upregulated in patients with active sarcoidosis (20, 48). The hallmark of the Th17 pathway is the production of IL-17. This proinflammatory cytokine with pleiotropic properties has been shown to be involved in the pathogenesis of various granulomatous diseases (49). Ten Berge et al. showed enhanced IL-17 expression in granulomas as well as increased numbers of IL-17 memory Th cells in the circulation and BALF of newly diagnosed sarcoidosis patients (50). Ostadkarampour et al. demonstrated the presence of T cells producing IL-17 in response to a mycobacterial antigen in patients with pulmonary sarcoidosis. The authors also observed higher levels of IL-17 and IL-17 producing cells in patients with Löfgren's syndrome suggesting a potential biomarker for the prognosis of sarcoidosis (51). Increased IL-17 responses might be related to aberrant metabolic pathways as shown by the abundant expression of hypoxia inducible factor (HIF) isoforms in granulomas and their association with Glut1 protein levels and enhanced IL-17 production. Downregulation of HIF in sarcoidosis peripheral blood mononuclear cells lead to a decrease in IL-17 production (52). An elevated expression of IL-17 receptor C on CD8^+^ T cells in peripheral blood was found in patients with ocular sarcoidosis (53).

Currently, three human monoclonal antibodies that target IL-17 have been approved for the treatment of psoriasis, psoriatic arthritis and axial spondyloarthritis (secukinumab, ixekizumab and brodalumab). In Crohn's disease, which is another disorder characterized by granuloma formation, IL-17 inhibition can exacerbate the inflammatory disease. In sarcoidosis patients, the role of IL-17 inhibition is less clear (54). Most of the available evidence on IL-17 blockade in patients with sarcoidosis comes from case reports. Previous reports suggest a favorable effect of secukinumab in two cases of sarcoidosis associated with TNF antagonists (55, 56). Conversely, our review of the literature identified two cases of sarcoidosis that either developed or worsened in patients taking secukinumab and ixekizumab (57, 58). However, a previous multicentre, randomized, double blind, phase II trial, assessing the efficacy and safety of secukinumab in non-infectious uveitis did not identify any safety concerns in the included patients with sarcoidosis (*n* = 4) (59). Further trials assessing the efficacy and safety of IL-17 blockade in sarcoidosis are warranted.

## Interleukin-12 and Interleukin-23 Blocking

Sarcoid granuloma formation is characterized by an influx of Th1 cells which spontaneously express IL-2 receptors and release interferon γ (IFN-γ), TNF-α, and IL-2 (5). IL-12 through its receptor (IL-12R) expressed on Th1 cells is one of the most important cytokines for inducing T cell response toward Th1 differentiation (60). Multiple studies have reported overexpression of IL-12 by activated alveolar macrophages in patients with active pulmonary sarcoidosis (61–65). IL-23 is a member of the IL-12 cytokine family which promotes Th17 responses through its receptor (IL-23R) (66). Transcriptomic analyses demonstrated upregulation of IL-23 in sarcoid lesions (67). IL-23R polymorphisms have also been shown to be implicated in the pathogenesis of sarcoidosis (68).

Ustekinumab is a monoclonal antibody that binds to the shared p40 unity of human IL-12 and IL-23, thus blocking Th1 and Th17 responses, respectively. It is approved for the treatment of psoriasis, psoriatic arthritis, Crohn's disease and ulcerative colitis (69). In a phase II, multicentre, randomized, double-blind, placebo-controlled trial (70), Judson et al. evaluated the safety and efficacy of ustekinumab in patients with chronic pulmonary and/or cutaneous sarcoidosis (70). Patients received ustekinumab 180 mg subcutaneously at week 0 and 90 mg at week 8, 16, and 24. Despite an excellent safety profile, ustekinumab failed to achieve the primary efficacy endpoint [change from baseline at week 16 in % predicted forced vital capacity (FVC%)] and there were no significant improvements in the major secondary endpoints. Guselkumab and risankizumab are specific anti-IL-23 monoclonal antibodies recently approved for the treatment of moderate-to-severe psoriasis. However, no data is currently available in patients with sarcoidosis. Similarly to IL-17 antagonists, several cases of induced or worsened sarcoidosis with ustekinumab or guselkumab have been reported in the literature (71–74).

## Cytotoxic T-lymphocyte Antigen 4 Blockade

Cytotoxic T-lymphocyte antigen 4 (CTLA-4) is a costimulatory molecule that is an important regulator of T cell activation and proliferation. CTLA-4 polymorphisms were shown to significantly influence phenotypes of sarcoidosis (75). Abatacept is a fusion protein of the extracellular domain of the CTLA-4 linked to a modified Fc of human immunoglobulin 1 (IgG1) inhibiting the activation of T cell responses (76). It is currently approved for the treatment of RA. To the best of our knowledge, there are no published studies or case reports concerning abatacept in sarcoidosis. We identified a prospective open-label trial (STAR trial NCT00739960) evaluating abatacept in refractory sarcoidosis. Unfortunately, this study has been terminated prematurely due to funding constraints.

## B-Cell Inhibition

Innate and T cell immunity play major roles in the pathogenesis of sarcoidosis. Several studies suggest a potential involvement of B cell immune responses in this disease (5, 77). Hypergammaglobulinemia and B cell accumulation in sarcoid lesions are frequently observed (78, 79). Saussine et al. showed increased levels of B-cell-activating factor (BAFF), also called BlyS (B lymphocyte stimulator), which is a cytokine involved in the survival and maturation of B cells in patients with active sarcoidosis (80). Rituximab (RTX) is an anti-CD20 monoclonal chimeric antibody that selectively depletes CD20^+^ B cell population. RTX was first approved for the treatment of non-Hodgkin B-cell lymphoma and later for rheumatoid arthritis and anti-neutrophil cytoplasmic antibody (ANCA) associated vasculitis. Despite several case reports of the effectiveness of RTX in patients with refractory sarcoidosis (81–90) data from clinical trials are scarce. In a prospective, open-label phase I/II trial, the authors assessed the effect of RTX in 10 patients with symptomatic moderate-to-severe pulmonary sarcoidosis refractory to corticosteroids plus one or more corticosteroid-sparing agents, including methotrexate and azathioprine (91). The authors observed very modest and inconsistent improvements in FVC% and 6-min walk test (6MWD) with only two patients having >10% improvement of FCV% and three patients having >50 m improvement in 6MWD by week 52. The prednisone doses were not changed during the study. RTX was generally well tolerated with only one patient hospitalized for pneumonia. Two patients died of respiratory failure linked to sarcoidosis progression. Belimumab, a human monoclonal antibody that inhibits BAFF/BlyS, is approved for the treatment of systemic lupus erythematosus and may represent a potential drug candidate for the management of refractory sarcoidosis. However, our literature review did not identify reports or ongoing trials concerning belimumab in sarcoidosis.

## Janus Kinase Inhibition

The activation of macrophages in sarcoid lesions is thought to be driven by Th1 immune responses and mediated by several cytokines including interferon-γ (IFN-γ) (92, 93). IFN-γ activates the Janus Kinase (JAK)-signal transducer and activator of transcription (STAT) signaling pathway which is involved in upregulating a set of genes involved in the inflammatory response (94). Transcriptomic analysis showed that JAK-STAT pathway activation signatures, especially STAT1 pathway, is activated in granulomatous diseases (95–98).

There are currently four JAK inhibitors available for clinical use: ruxolitinib for the treatment of myeloproliferative disorders; tofacitinib for rheumatoid arthritis (RA), psoriatic arthritis and ulcerative colitis; and baricitinib, upadacitinib and filgotinib for RA. Our literature review identified 7 reports concerning JAK inhibitors use (ruxolitinib *n* = 3; tofacitinib *n* = 3; baricitinib *n* = 1) in cases of refractory sarcoidosis. Ruxolitinib, a JAK 1 and 2 inhibitor, improved multivisceral involvement in three patients with sarcoidosis among whom two were treated for concomitant polycythemia vera (99–101). Damsky et al. reported dramatic positive effects of tofacitinib, a JAK 1 and 3 inhibitor, in a patient with severe cutaneous sarcoidosis refractory to topical and systemic corticosteroids and multiple corticosteroid-sparing agents including minocycline, hydroxychloroquine, methotrexate, tacrolimus and adalimumab. During treatment, the authors observed a downregulation of JAK/STAT signature on skin samples (102). The positive effect of tofacitinib was also observed in four patients with refractory cutaneous sarcoidosis (*n* = 3) and granuloma annular (*n* = 1) (103). Tofacitinib resulted in a mean improvement in clinical activity and histologic resolution was documented in all patients. The same group reported another case of severe multiorgan sarcoidosis involving the lungs, lymph nodes, bones, and skin, not controlled with prednisone, mycophenolate sodium, methotrexate, infliximab, rituximab and intravenous immunoglobulins (104). In this patient, tofacitinib treatment resulted in clinical remission of cutaneous lesions as well as resolution of positron emission tomography hypermetabolized lesions in internal organs after 6 months. Scheinberg et al. reported the case of a 35-year-old female patient with fever, arthralgia and hilar and cervical adenopathy revealing sarcoidosis initially treated with prednisone (105). Due to persistent daily low grade fever and arthralgia she was started on baricitinib, a selective JAK 1 and 2 inhibitor, with complete resolution of clinical symptoms and lymph node disease after week 12. Despite these encouraging data, the safety profile of JAK inhibitors in sarcoidosis remains to be confirmed. There are currently two ongoing open-label trials evaluating tofacitinib 5 mg twice daily as a corticosteroid-sparing agent in pulmonary sarcoidosis (NCT03793439) and in the treatment of cutaneous sarcoidosis and granuloma annular (NCT03910543).

## Phosphodiesterase Type 4 Inhibition

Inhibition of PDEA4 prevents cyclic AMP (cAMP) being hydrolysed to AMP resulting in increased intracellular levels of cAMP and inhibiting the expression of proinflammatory cytokines (106). Apremilast is an orally administered phosphodiesterase type 4 (PDEA4) inhibitor approved for the treatment of psoriasis and psoriatic arthritis. Baughman et al. reported the effects of apremilast in 15 patients with active cutaneous sarcoidosis (107). The authors observed significant improvements in skin scores in 14 patients treated with apremilast 20 mg twice daily. Three patients developed worsening of cutaneous sarcoid lesions within 3 months after discontinuation of apremilast. A phase 2 and 3 open-label trial evaluating the efficacy and safety of apremilast in chronic cutaneous sarcoidosis (NCT00794274) has been completed; however the results are not yet available.

## Conclusion

Refractory sarcoidosis, broadly defined as failure to attain clinical remission after appropriate treatment with corticosteroids and conventional immunosuppressant, is associated with increased morbidity and mortality. At present, TNF antagonists can be considered standard-of-care therapy in severe cases of refractory sarcoidosis. However, treatment options in patients with multidrug-refractory sarcoidosis failing initial anti-TNF therapy is a challenging issue in clinical practice. In the clinical practice, assessing adherence, ruling out differential diagnosis, checking anti-TNF blood concentrations and testing for anti-drug antibodies, should be done before classifying patients as refractory and intensifying treatment. In this setting, new potential useful agents including IL-6, IL-17, and JAK inhibitors with evidence consisting largely of observation or uncontrolled studies are the most promising ones. Data from ongoing prospective clinical trials should give further information on clinical effects of these agents in refractory sarcoidosis.

## Author Contributions

All authors were involved in drafting the article or revising it critically for important intellectual content, and approved the final version to be submitted for publication. PC had full access to all of the data in the study and takes responsibility for the integrity of the data and the accuracy of the data analysis.

## Conflict of Interest

PC has received consulting and lecturing fees from Abbvie, Alnylam, Bristol-Myers Squibb, Gilead, Glaxo Smith Kline, Innotech, Janssen, Servier and Vifor. DS has received consulting and lecturing fees from from Medimmune, Abbvie, Bristol Meyer Squibb, Celgene, Sanofi, Roche, Servier, Gilead, AstraZeneca and Glaxo Smith Kline. The remaining authors declare that the research was conducted in the absence of any commercial or financial relationships that could be construed as a potential conflict of interest.
