# Supplementary material for: Emerging Molecular Targets for the Treatment of Refractory Sarcoidosis
Source: Front Med (Lausanne). 2020 Nov 24;7:594133. doi: 10.3389/fmed.2020.594133 (PMC7732552; doi:10.3389/fmed.2020.594133)
Supplement: Supplementary file 1 [file Table_1.docx]

| **Supplementary Table 1**. Summary of reported cases in the literature of the use of targeted biologic and synthetic agents (other than TNF inhibitors) in the setting of sarcoidosis. | | | | |
| --- | --- | --- | --- | --- |
| **Case reports** | **Target** | **Drug** | **Efficacy (Y/N)** | **Outcome** |
| Awano N [29] | IL-6 | **Tocilizumab** | **Y  (Multicentric Castlemans's  disease)** | Clinical improvement of Multicentric Castleman's disease in a 60-year-old woman with pre-existing sarcoidosis |
| Semiz H [30] | IL-6 | **Tocilizumab** | **Y  (adult onset Still disease)** | Clinical improvement of adult onset Still disease in a 24-year-old man with pre-existing sarcoidosis |
| Sharp M [31] | IL-6 | **Tocilizumab** | **Y** | Improved symptoms and organ function in 4 patients with refractory chronic severe sarcoidosis |
| Nutz A [35] | IL-6 | **Tocilizumab** | **N  (induced sarcoidosis)** | Onset of sarcoidosis in a patient with rheumatoid arthritis |
| Bustamante L [36] | IL-6 | **Tocilizumab** | **N  (induced sarcoidosis)** | Onset of sarcoidosis in a patient with rheumatoid arthritis |
| Shono Y [37] | IL-6 | **Tocilizumab** | **N  (induced sarcoidosis)** | Onset of cutaneous sarcoidosis in a patient with rheumatoid arthritis |
| Del Giorno R [38] | IL-6 | **Tocilizumab** | **N  (induced sarcoidosis)** | Onset of cutaneous sarcoidosis in a patient with giant cell arteritis |
| Toussirot E [55] | IL-17 | **Secukinumab** | **Y** | Improvement of TNF inhibitor-induced sarcoidosis-like reaction in a patient with axial spondyloarthritis |
| Eichhoff G [56] | IL-17 | **Secukinumab** | **Y** | Improvement of TNF inhibitor-induced sarcoidosis-like reaction in a patient with psoriasis |
| Nyckowski T [57] | IL-17 | **Secukinumab** | **N  (induced sarcoidosis)** | Onset of sarcoidosis in a patient with psoriatic arthritis |
| Sambharia M [58] | IL-17 | **Ixekizumab** | **N** | Acute worsening of pulmonary sarcoidosis in a patient with psoriasis |
| Powell JB [71] | IL-12/23 | **Ustekinumab** | **N  (induced sarcoidosis)** | Onset of sarcoidosis in a patient with plaque psoriasis |
| Gad MM [72] | IL-12/23 | **Ustekinumab** | **N  (induced sarcoidosis)** | Onset of sarcoidosis in a patient with refractory psoriasis |
| Kobak S [73] | IL-12/23 | **Ustekinumab** | **N  (induced sarcoidosis)** | Onset of sarcoidosis in a patient with psoriatic arthritis |
| Thomas AS [74] | IL-23 | **Guselkumab** | **N** | Uveitis worsening in a patient with sarcoidosis |
| Beccastrini E [81] | CD20 | **Rituximab** | **Y** | Ocular improvement in a patient with severe sarcoid panuveitis |
| Cinetto F [82] | CD20 | **Rituximab** | **Y** | Clinical improvement in 3 patients with refractory sarcoidosis |
| Gottenberg JE [83] | CD20 | **Rituximab** | **Y** | Clinical improvement in a patient with lymph node sarcoidosis |
| Zella S [84] | CD20 | **Rituximab** | **Y** | Clinical improvement in 3 patients with probable neurosarcoidosis |
| Bomprezzi R [85] | CD20 | **Rituximab** | **Y** | Cinical improvement in a patient with neurosarcoidosis |
| Sawaya R [86] | CD20 | **Rituximab** | **Y** | Visual acuity improvement in patient with sarcoidosis associated with neuromyelitis optica |
| Lower EE [87] | CD20 | **Rituximab** | **Y** | Improvement in 4 patients with ocular sarcoidosis |
| Dalia T [88] | CD20 | **Rituximab** | **Y** | Sarcoidosis with cutaneous medium-vessel granulomatous vasculitis successfully treated with Rituximab |
| Earle B [89] | CD20 | **Rituximab** | **Y** | Ocular improvement in 11-year-old patient with optic neuritis secondary to neurosarcoidosis |
| Krause ML [90] | CD20 | **Rituximab** | **Y** | Improvement of refractory cardiac sarcoidosis with life-threatening arrhythmia |
| Rotenberg C [99] | JAK 1/2 | **Ruxolitinib** | **Y** | Improvement of refractory sarcoidosis in a patient with associated JAK2-mutated polycythemia vera |
| Levraut M [100] | JAK 1/2 | **Ruxolitinib** | **Y** | Clinical and biological remission in a patient with refractory sarcoidosis-like systemic granulomatosis |
| Wei JJ [101] | JAK 1/2 | **Ruxolitinib** | **Y** | Resolution of cutaneous sarcoidosis in a patient with concomitant polycythemia vera |
| Damsky W [102] | JAK 1/3 | **Tofacitinib** | **Y** | Improvement of skin lesions in a patient with refractory cutaneous and pulmonary sarcoidosis |
| Damsky W [103] | JAK 1/3 | **Tofacitinib** | **Y** | Improvement in the Cutaneous Sarcoidosis Activity and Morphology Instrument in 3 patients with recalcitrant cutaneous sarcoidosis |
| Damsky W [104] | JAK 1/3 | **Tofacitinib** | **Y** | Clinical remission of cutaneous sarcoidosis and resolution of positron emission tomography avid lesions in internal organs |
| Scheinberg M [105] | JAK 1/2 | **Baricitinib** | **Y** | Rapid resolution of clinical symptoms in a p+A1:E34atient with multisystem sarcoidosis |
| IL, interleukin; CD20, cluster of differentiation 20; JAK, Janus kinase. | | | | |
